# Effect of *Alcaligenes* sp. on corrosion behavior of X65 steel in simulated offshore oilfield-produced water

**DOI:** 10.3389/fmicb.2023.1127858

**Published:** 2023-03-17

**Authors:** Peiyu Shi, Min Du, Jian Wang

**Affiliations:** Key Laboratory of Marine Chemistry Theory and Technology, College of Chemistry and Chemical Engineering, Ministry of Education, Ocean University of China, Qingdao, China

**Keywords:** X65 steel, *Alcaligenes* sp., proteoglycan, corrosion inhibition, biofilm

## Abstract

In this paper, the effect of *Alcaligenes* sp. on the corrosion process of X65 steel was investigated by using non-targeted metabolomics techniques for comprehensive characterization of metabolites, combined with surface analysis techniques and electrochemical testing. The results showed that the organic acids produced by *Alcaligenes* sp. accelerated the corrosion process of X65 steel in the early stage, and the presence of *Alcaligenes* sp. promoted the deposition of stable corrosion products and minerals in the middle and late stages. In addition, proteoglycans and corrosion inhibiting substances were enriched on the metal surface, which enhanced the stability of the film. The combined effect of multiple factors makes the mixed film of biofilm and corrosion products more dense and complete, which effectively inhibits the corrosion of X65 steel.

## 1. Introduction

X65 steel is widely used in the development of oil and gas fields because of its good corrosion resistance, high strength and excellent resistance to fracture toughness. However, X65 steel faces serious corrosion problems in the process of actual use. The global economic loss caused by steel corrosion is as high as $700 billion every year, among which the corrosion damage caused by the marine environment seriously restricts the development of the marine economy (Hou and Lu, 2018). Microorganisms are one of the very important factors affecting the corrosion of metallic materials. The microbial corrosion (MIC) accounts for 20% of the corrosion damage of metals and construction materials(Li et al., 2001). And it often works synergistically with other corrosion processes such as stress corrosion, crevice corrosion, etc., causing huge safety hazards.

There is a wide range of microorganisms, of which sulphate-reducing bacteria (SRB) cause the most serious MIC, and almost all metals and their alloys are affected by SRB corrosion (Videla, 2000). As research progressed, researchers found that many bacteria inhibited corrosion of metals, such as *Pseudomonas*, *Serratiamarcescens*, *Shewanella algae*, *Pseudomonas flava* and *Bacillus*, etc. (Qu et al., 2015; Guo et al., 2021; Gao et al., 2022). The inhibition mechanism includes the shielding effect of biofilm and corrosion products, inhibition of the secretion of corrosive substances, and alteration of the local microenvironment (Wang et al., 2022). The widespread use of chemical fungicides to control the harm caused by microbial corrosion, although a certain effect, but the long-term use of fungicides will make the bacteria resistant, and at the same time will bring pollution to the environment (Enning et al., 2016). Microbial control method mainly uses the symbiotic, competitive or antagonistic effect between different populations to inhibit the corrosion of harmful microorganisms through the transformation of the dominant population. It is capable of autonomous regulation and intelligent protection, and has the characteristics of long-term inhibition and environmental friendliness, etc. It has very important economic and social benefits for the prevention and control of microbial corrosion and for the protection of the environment. At present, the use of microorganisms to inhibit microbial corrosion research is in the initial stage, for the selection of strains and their participation in the mechanism of corrosion electrochemistry is not fully understood, is not enough to guide the practical application. But it has a great prospect of application as an efficient and environmentally friendly suppression method (Lou et al., 2021).

*Alcaligenes* sp. are Gram-negative bacteria that are widely distributed in nature and exist in water, soil, and plants and animals (Lei et al., 2011; Lu et al., 2012). *Alcaligenes* sp. can degrade many toxic substances such as phenol, cyanide, arsenite, hydrogen sulfide (Rattanapan et al., 2010; Sun et al., 2017; Yin et al., 2017; Sha’arani et al., 2019). The peptide compounds, terpenoids and glycosides isolated from the metabolites produced by *Alcaligenes* sp. have applications in antimicrobial and provide a reference for microbial control and antibiotic development (Kapley et al., 2013, 2016; Yokoyama et al., 2013). *Alcaligenes* sp. are also an important source for some enzyme research (Bhatia et al., 2014; Nataningtyas et al., 2019). The use of *Alcaligenes* sp. allows for large-scale production and fermentation of key enzymes in antibiotic production (Wang et al., 2006). *Alcaligenes* sp. can produce chitinases, which can convert chitin into oligosaccharides, and these degradation products have antitumor and antifungal activities (Kavroulakis et al., 2010; Abraham et al., 2015). In addition, *Alcaligenes* sp. inhibit the overgrowth of organisms such as algae (Xue et al., 2022). It can be seen that *Alcaligenes* sp. not only have a wide range of sources, but also produce a variety of diverse and complex secondary metabolites. These substances have applications in antibiotic development and biological control. However, the effects of *Alcaligenes* sp. and their metabolites on metal corrosion or inhibition are not well understood and have been rarely reported in the field of metal corrosion control. Due to the complex composition of the marine environment, high salt water, extreme temperature and pressure, and special light characteristics, marine microorganisms have multiple pathways of metabolism, and therefore marine microorganisms can produce a variety of special products that are different from those of terrestrial origin. We screened a strain of *Alcaligenes* sp. from marine krill and, after preliminary tests, found that the strain had inhibitory effects on the growth of SRB in oilfield produced water and on the corrosion of carbon steel by SRB in oilfield produced water. To study the effect of this strain on the corrosion morphology and corrosion product composition of metals, to study the evolution of its metabolites on the metal surface and the effect on the electrochemical process of corrosion at different periods of bacterial growth, and to take a comprehensive view of the effect of *Alcaligenes* sp. on metals. It is beneficial to further broaden the application field of *Alcaligenes* sp., and it is of reference guidance to clarify the mechanism of action and application value of *Alcaligenes* sp. in the field of metal corrosion prevention and control, and to provide guidance for the subsequent research on the inhibition mechanism of *Alcaligenes* sp. on SRB corrosion.

In this paper, the corrosion behavior of *Alcaligenes* sp. on X65 steel was studied by comparing the *Alcaligenes* sp. medium with the sterile medium in simulated oilfield produced water, and the corrosion behavior of *Alcaligenes* sp. on X65 steel was analyzed by weight loss method and laser confocal scanning microscopy technique. The growth of microorganisms was determined by dilution plate counting method, focusing on the difference between the initial, intermediate and final stages of growth. The composition of metabolites of *Alcaligenes* sp. was determined by non-targeted metabolomics techniques, and the effects of *Alcaligenes* sp. and their metabolites on the corrosion process of X65 steel were investigated by combining scanning electron microscopy observation, fluorescence-laser confocal microscopy techniques, Raman spectroscopy testing techniques and electrochemical testing techniques.

## 2. Materials and methods

### 2.1. Experimental material preparation

In this paper, electrochemical and corrosion characterization experiments were conducted using X65 steel produced by Shandong Yangxin Shengxin Technology Co. The main composition of X65 steel (quality fraction/%): 0.03C, 1.51Mn, 0.17Si, 0.02P, 0.17Ni, 0.04Cu, 0.16Mo, 0.06Nb, 0.02Al, 0.01Ti, and the remaining amount of Fe. Specimens of size 10 mm × 10 mm × 3 mm were used for electrochemical testing. One end of the specimen was soldered to the copper wire with tin wire, leaving a 1 cm^2^ end surface as the working surface, and the rest was sealed by filling with epoxy resin to make the specimen into an electrode. The specimens used for the loss-in-weight method were 10 mm × 10 mm × 3 mm, with a small hole of about 6 mm in diameter at one end. All specimens were polished with 400#, 800#, 1,000# and 2000# water-grit sandpaper until the surface was smooth and mirror-like. Then they were degreased with anhydrous ethanol and rinsed with distilled water and dried in high purity N_2_ (99.999%). All X65 steel specimens were irradiated with a UV lamp in a sterile bench for 30 min prior to use to prevent contamination of the specimen surface with other bacteria.

### 2.2. Bacterial enumeration and medium pH detection

The experimental strain was the *Alcaligenes* sp. isolated from Antarctic krill. The medium composition of the *Alcaligenes* sp. was 20 g/l glucose, 10 g/l starch, 10 g/l yeast extract, 10 g/l peptone, 3 g/l beef paste, 2 g/l CaCO_3_, 0.5 g/l KH_2_PO_4_, 0.5 g/l MgSO_4_ and 1 l seawater. *Alcaligene sp.* were subjected to shake flask culture: liquid medium, selected activated single colonies were inoculated in the medium and incubated at 28°C, 200 r/min in a constant temperature shaker for 3 days to obtain a culture for inoculation. The experimental medium was 400 ml of simulated oilfield produced water plus 100 ml of *Alcaligenes* sp. medium, and the composition of simulated oilfield produced water was 0.0322 g/l Na_2_CO_3_, 3.0638 g/l NaHCO_3_, 1.1096 g/l Na_2_SO_4_, 0.5053 g/l CaCl_2_, 1.6603 g/l MgCl_2_, 0.3553 g /L KCl, 16.617 g/l NaCl, 1 l distilled water. The number of planktonic and sequestered bacteria in the medium was measured by plate counting method and the growth curve was plotted. To perform the count, the sample to be tested is first diluted in a gradient, and the appropriate dilution is selected for coating. The autoclaved medium is poured into sterile plates while still hot and numbered. When sampling the sessile bacteria, the corrosion products on the surface of the specimen need to be scraped off with a sterilized brush, and then the biofilm on the surface is scraped in a sterile sampling tube and diluted step by step with sterile water according to the gradient dilution method. It is worth noting that in the process of dilution, the liquid in the sampling tube is repeatedly pumped several times with a pipette to make the solid bacteria in sterile water dispersion. Then inoculate 0.1 ml of bacterial solution sequentially on agar plates by pipetting gun, where each dilution was tested 3 times in parallel, and use the applicator to spread the bacterial solution evenly on the plates. The coated plate was left on the table for 20–30 min so that the bacterial solution could penetrate into the medium, inverted the plate, sealed with sealing film, and incubated at constant temperature until the colonies grew and counted. While the bacteria were counted, 5 ml of the solution was removed daily and the change of pH value in the medium was measured using a pH meter.

### 2.3. Loss-in-weight test

The average corrosion rate was determined by the loss-in-weight method at three different stages (7, 14, and 21 days) of growth of *Alcaligenes* sp. in the produced water of the simulated oil field. The specimens used were 50 mm × 10 mm × 3 mm, and the temperature of the loss-in-weight experiments was controlled at 25°C. In order to ensure the accuracy of the data, three pieces of X65 steel were selected for each group of experiments, and the weight loss rates of the three pieces were averaged. Before conducting the weight loss test, the weight of the specimen was recorded on an electronic analytical balance as $m_{1}$. X65 steel was soaked in the experimental medium for various times and then removed, the surface of the specimen was rinsed with distilled water, the loose corrosion products were gently brushed off with a brush, then the specimen was placed in a pickling solution containing hexamethyl tetramine corrosion inhibitor, the corrosion products were removed, the specimen was rinsed in turn with distilled water and anhydrous ethanol and finally dried with N_2_. Weigh, remove the corrosion products, weigh in, repeat several times until the weight difference between the two times after removing the corrosion products is not significant, and record the data as $m_{2}$ (ASTM, 2011). Calculate the corrosion rate (ν) of the specimen under experimental conditions according to Equation (1):


(1) $$v=\frac{m_{1}-m_{2}+\Delta m}{st}\left(\frac{g}{m^{2}}\cdot h\right),$$

$m_{1}$ is the mass of the initial sample of the weightlessness experiment (g); $m_{2}$ is the mass of the final sample of the weightlessness experiment (g); Δm is the reduced mass of the sample in the previous time period of the weightlessness experiment (g); t is the experimental immersion period (h); ν is the corrosion rate of the specimen in the corrosion solution (g/m^2^ h).

### 2.4. Surface observation and analysis

The surface of the specimen with corrosion products removed was observed using a laser confocal scanning microscope (CLSM, Keyence, VK-X200). Before conducting the test, X65 steel was soaked in the experimental medium for various times and then removed, the surface of the specimen was rinsed with distilled water, the loose corrosion products were gently brushed off with a brush, and then the specimen was soaked in a pickling solution containing hexamethyltetramine corrosion inhibitor for 5 min. After the rust removal was completed, the surface of the specimen was rinsed with distilled water and anhydrous ethanol in turn, dried in N_2_, and vacuum sealed in a bag for observation.

The biofilm and corrosion products on the sample surface were observed using a scanning electron microscope (SEM, Gemini 300, Zeiss). A standard phosphate buffer solution (PBS) was prepared and then sterilized, followed by rinsing the sample surface with the solution in order to remove any unattached debris from the surface. The samples were then fixed in a 5% glutaraldehyde solution (diluted with sterilized PBS) for 2 h. After fixation, the samples were dehydrated in steps of 30, 50, 70, 90, and 100% anhydrous ethanol for 15 min and dried under natural conditions before SEM surface microscopic testing.

The surface corrosion product composition was analyzed using Raman spectroscopy (DXR Microscope). Before testing, the specimens were first removed from the experimental medium, rinsed well using sterilized PBS, dried in N_2_ and then vacuum-encapsulated.

The distribution of live cells, dead cells and EPS (proteins, polysaccharides) on the surface of the specimens was examined using a fluorescent laser confocal microscope (Olympus FV1200). The live and dead bacteria were stained with SYTO-9 dye and propidium iodide dye (Invitrogen, Eugene, OR, United States), respectively. The excitation wavelengths used were 488 and 559 nm, respectively. Finally, a high-resolution 3D fluorescence image was obtained to demonstrate the biofilm viability. To further analyze the distribution of the proteins and polysaccharides, the proteins and polysaccharides were stained with fluorescein isothiocyanate (FITC) and iFluor 555-concanavalin A (ConA), respectively (Adav et al., 2010). Then, the stained coupons were analyzed using CLSM.

### 2.5. Non-targeted metabolomics assay

*Alcaligenes* sp. were inoculated in sterilized liquid medium and incubated at 28°C for 14d in a constant shaker at 200 r/min. The supernatant was obtained by centrifugation and filtration, 1 ml of supernatant was taken, the same volume of extracted methanol acetonitrile solution (1:1, *v*/*v*) was added, vortexed for 60 s, sonicated at low temperature for 30 min, twice, placed at −20°C for 1 h to precipitate the protein, centrifuged at 12,000 rpm, 4°C for 20 min, the supernatant was freeze-dried and re-dissolved in 200 μl of 30% ACN, vortexed, centrifuged at 14,000 *g* for 15 min at 4°C, and the supernatant was taken on the machine for detection. The metabolites in the samples were detected by ultra performance liquid chromatography-tandem time of flight mass spectrometry (UHPLC-QE), mainly consisting of ultra performance liquid chromatography (Vanquish, UPLC, Thermo, United States) and high resolution mass spectrometry (Q Exactive, Thermo, United States). Raw data were processed with metabolomics software Progenesis QI (Waters Corporation, Milford, United States) for baseline filtering, peak identification, integration, retention time correction, and peak alignment, resulting in a data matrix of retention time, mass-to-charge ratio, and peak intensity, which was matched with information from local databases as well as commercial databases for the detection of metabolites in samples.

### 2.6. Electrochemical methods

The electrochemical impedance spectroscopy (EIS) and potentiodynamic polarization (PDP) curves were performed using a Gamry electrochemical workstation (Reference 600, Gamry Instrument Inc., Warminster, PA, United States). A three-electrode medium was used for all electrochemical tests: X65 steel as the working electrode, saturated glycerol electrode (SCE) as the reference electrode, and noble metal oxide electrode (MMO) as the counter electrode. The frequency of the EIS measurements ranged between 10^−2^ to 10^5^ Hz with an amplitude of 10 mV in relation to the open circuit potential (OCP). By establishing a suitable equivalent circuit model, the EIS data were analyzed with Zview2 software (Scribner Inc.) to obtain useful interface electrochemical parameters. Potentiodynamic polarization curves were scanned in the potential range of − 200 to + 300 mV vs. OCP at a scan rate of 0.5 mV/s. And the polarization curves were analyzed using Cview2 software (Scribner Inc.).

## 3. Results

### 3.1. Bacterial growth and weight loss results

**Figure 1:**
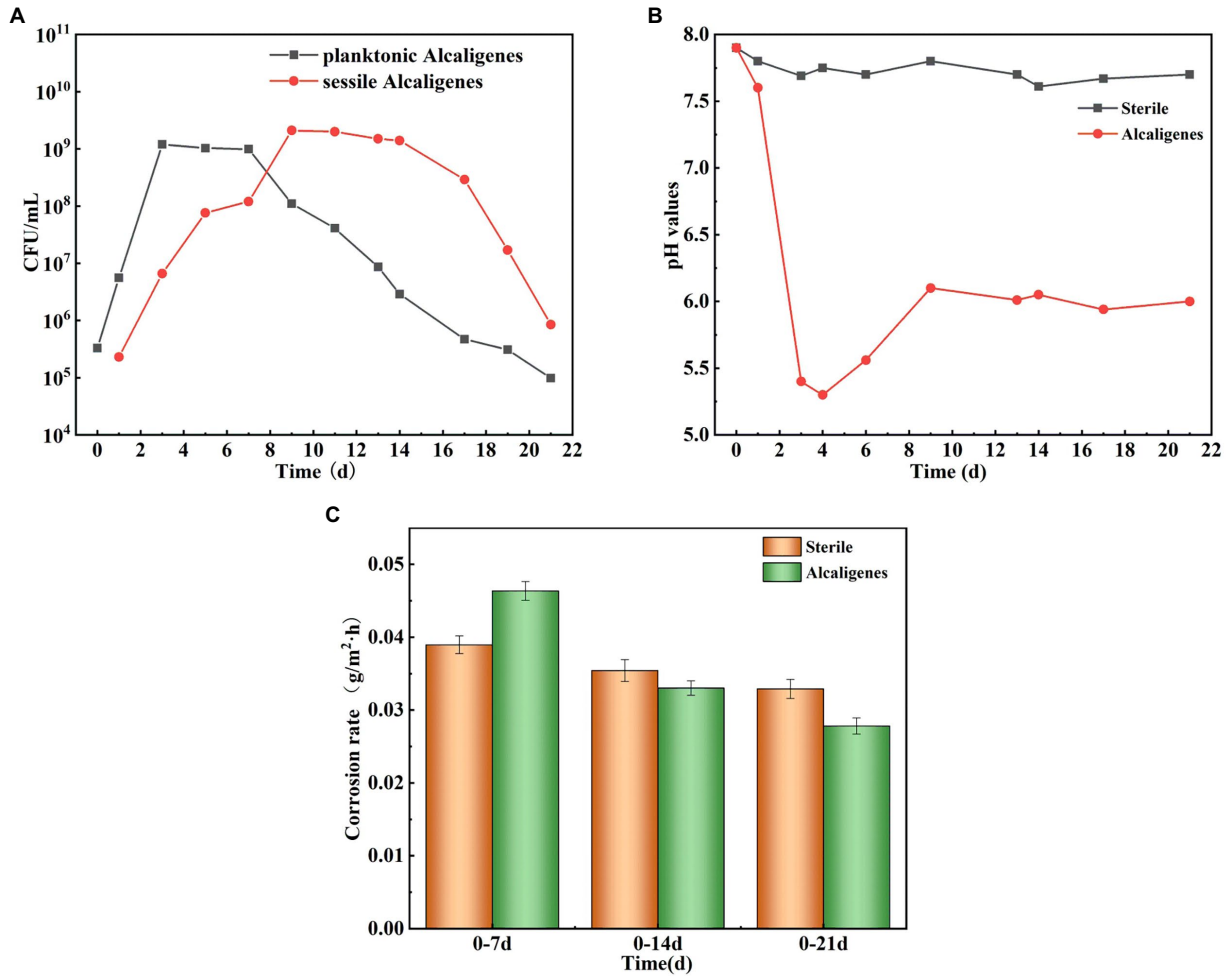
**(A)** Growth curves of *Alcaligenes* sp. in planktonic and sessile bacteria; **(B)** Trend of pH over time in sterile and *Alcaligenes* sp. media; **(C)** Average corrosion rate of X65 steel in sterile and *Alcaligenes* sp. media.

Figure 1A shows the growth curves of *Alcaligenes* sp. in the simulated oilfield produced water for planktonic and sessile bacteria. At the beginning of the experiment, the number of *Alcaligenes* sp. inoculated in the solution was 3.3 × 10^5^ CFU/ml, and it can be seen that the growth curves of both showed a growth-stabilization-decrease pattern. The number of planktonic bacteria in the solution reached the maximum value of 1.2 × 10^9^ CFU/ml in 3d. During the 4–7 days period of stable growth, the bacterial population grew slowly and without significant order of magnitude changes. Seven days later, the number of *Alcaligenes* sp. started to decrease gradually and entered the decay period. The number of sessile bacteria was low in the first 1–2 days, and then increased rapidly. The maximum value of 2.1 × 10^9^ CFU/ml was reached at the ninth day. The number of sessile bacteria remained relatively stable from 9 to 14 days, and then gradually decreased. Compared to planktonic bacteria, the extreme value of the number of sessile bacteria appeared later, which is due to the need for some dissolved organic and inorganic particles to adsorb to the surface of the material, forming a conditioned film favorable for bacterial adsorption and growth on the surface of the material, followed by bacterial transfer to the metal surface (Hou et al., 2017). In the later period, due to the consumption of nutrients by *Alcaligenes* sp., the nutrient content of the medium was insufficient and the bacterial population gradually decreased. pH is an important factor affecting the growth of *Alcaligenes* sp. and the adhesion and complexation of EPS on metal surfaces, and also the metabolic activities of *Alcaligenes* sp. changes the pH of the environment. Figure 1B shows the change in pH over time in the sterile and the medium containing *Alcaligenes* sp. The results show that the pH values in the sterile medium were relatively stable, with minimal changes in pH over time days. In the *Alcaligenes* sp. medium, the pH decreased rapidly from 7.9 to 5.3 during 0–4 days due to the increasing number of *Alcaligenes* sp. and their metabolic activity, which produced organic acids by consuming organic matter in solution (Yang et al., 2019). The pH increases during 4–9 days, which is related to the stabilization of the bacterial population in solution. After 9 days the pH remains relatively stable, which is due to the decay phase of *Alcaligenes* sp., with a gradual increase in the number of bacterial deaths, a gradual decrease in the total population, and a decrease in metabolic activity. In order to investigate the effect of *Alcaligenes* sp. on the corrosion rate of X65 steel, the average corrosion rate of X65 steel in *Alcaligenes* sp. and sterile media at different periods was calculated using the weightless hanging method. From Figure 1C, we can get that the average corrosion rate of the *Alcaligenes* sp. medium and the sterile medium in the simulated oilfield produced water environment showed a general decreasing trend, and *Alcaligenes* sp. medium corrosion rate decreased significantly. The average corrosion rate of steel in the *Alcaligenes* sp. medium was 0.046 g/m^2^ h in 0–7 days, which was about 1.2 times of that in the sterile medium. With the extension of the immersion time, the average corrosion rate of steel in the *Alcaligenes* sp. medium in 0–14 days decreased to 0.033 g/m^2^ h, slightly lower than the sterile medium. 14–21 days average corrosion rate of steel continued to decrease. By 14–21 days, it decreased to 0.027 g/m^2^ h, and the average corrosion rate in this time period was about 0.8 times that of the sterile medium.

### 3.2. Corrosion morphology analysis

**Figure 2:**
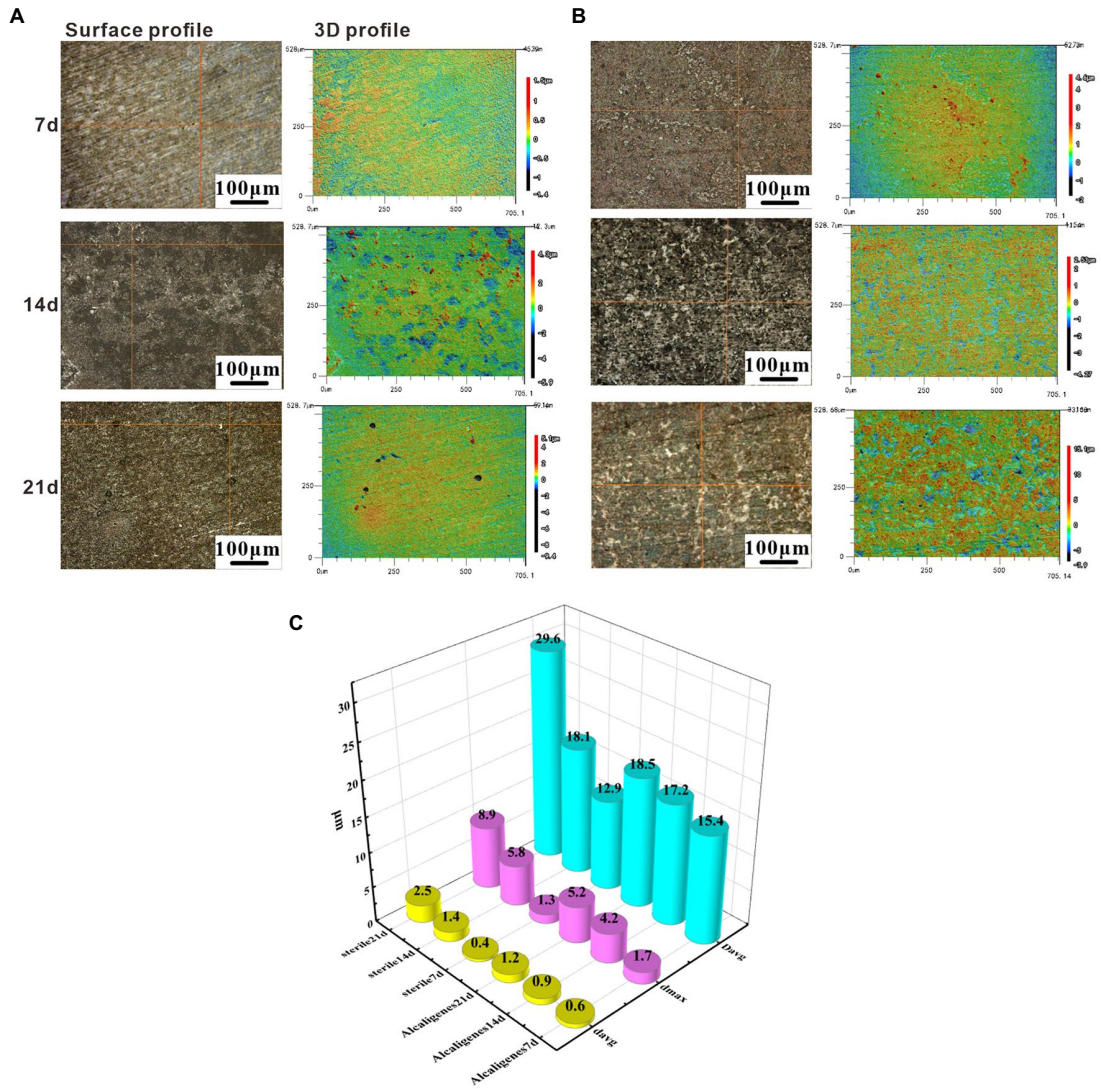
Removing surface corrosion products of X65 steel after immersion in **(A)** sterile and **(B)** *Alcaligenes* sp. media for different periods laser confocal scanning microscope (CLSM) image: surface topography and 3D profile. **(C)** Variation of average pitting parameters for X65 steel immersed in sterile and *Alcaligenes* sp. media for different periods.

To investigate the effect of *Alcaligenes* sp. on the corrosion of X65 steel, the local corrosion parameters of the steel in the sterile and *Alcaligenes* sp. media were measured separately. Figure 2A shows CLSM images of X65 steel with corrosion products removed after 7, 14, and 21 days immersion in sterile medium, including the surface morphology and 3D profile of X65 steel. Figure 2B shows CLSM images of X65 steel with corrosion products removed after 7, 14, 21 days immersion in *Alcaligenes* sp. medium. By comparing Figures 2A,B can be seen, clear scratches can be seen on the surface of carbon steel in the sterile medium at 7 days, and occasional pits on the surface of carbon steel in the *Alcaligenes* sp. medium at 7 days, and the local corrosion is not obvious. At 14 days, the surface scratches on the specimen are blurred, the roughness increases, the depth of corrosion pits increases significantly, and the surface is dominated by round-shaped corrosion pits in the sterile medium, and the pits grow significantly along the transverse direction, showing a bar shape, and the growth along the longitudinal direction is not obvious in the *Alcaligenes* sp. medium. At 21 days, there were obvious pits, the pits were randomly distributed, and the depth of the pits increased further in the sterile medium. Although the pits developed along the longitudinal direction in the *Alcaligenes* sp. medium, the decline was much smaller than in the sterile medium, and the corrosion process was obviously inhibited.

Figure 2C shows the variation of the average pitting parameters (average corrosion pit depth *d*_avg_, maximum corrosion pit depth *d*_max_, and average corrosion pit diameter *D*_avg_) for X65 steel in the sterile and *Alcaligenes* sp. media over time, and differences in the rate of pitting development can be observed at different times in the different media. At 7 days, the differences in *d*_max_, *D*_avg_, and *d*_avg_ were not significant in the sterile and *Alcaligenes* sp. media, and the local corrosion was not significant. At 14 days, the *d*_max_ of the specimen from the sterile medium was approximately 1.4 times that of the *Alcaligenes* sp. medium. At 21 days, the *d*_max_, *D*_avg_, and *d*_avg_ of the specimens from the sterile medium were deeper than those from the *Alcaligenes* sp. medium, and the *d*_max_ of the specimens from the sterile medium was about 1.7 times higher than that of the *Alcaligenes* sp. medium.

### 3.3. Morphology and composition characterization of biofilms and corrosion products

#### 3.3.1. Surface morphology observation

**Figure 3:**
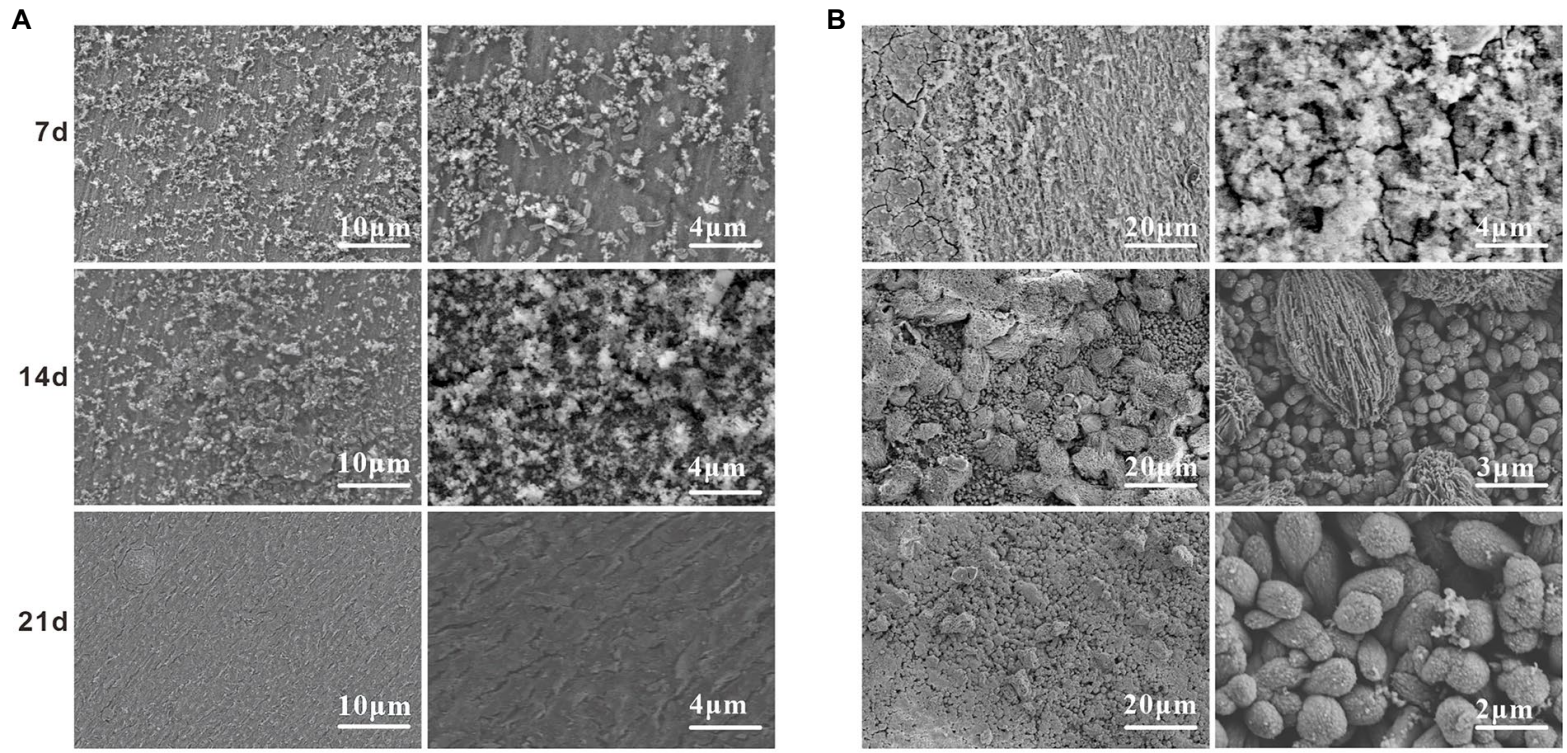
scanning electron microscope (SEM) images on the surface of X65 steel after different periods of immersion in **(A)** sterile and **(B)** *Alcaligenes* sp. media.

Scanning electron microscope surface microscopic tests were conducted on the specimens in the sterile and *Alcaligenes* sp. media to compare the two media and to analyze the role of the morphology and denseness of the biofilm and corrosion products in the corrosion process of *Alcaligenes* sp. Figure 3A shows the corrosion morphology of X65 steel in the sterile medium. At 7 days, the surface of the specimen is distributed with cotton wool-like loose corrosion products. At 14 days, the surface of carbon steel of the sterile medium is divided into two layers inside and outside, the inner layer is a layer of corrosion products formed by spherical corrosion products, the outer layer is distributed with sparse volume of loofah-like corrosion products, and there are large gaps between the corrosion products. At 21 days the granular corrosion products join to form a complete and dense film layer, which can prevent the diffusion of corrosive ions from the solution to the surface of the substrate, providing a degree of protection for X65 steel, resulting in a reduction in the corrosion rate. Figure 3B shows the corrosion morphology of X65 steel in the *Alcaligenes* sp. medium, which differs from the sterile medium, indicating that *Alcaligenes* sp. has some influence on the formation of corrosion products. At 7 days, the corrosion products are sparsely distributed on the surface of the specimen and the bare metal matrix can be seen. And in 0–7 days, the bacterial metabolism is more vigorous, the organic acid produced, the surface film layer of unevenness and incompleteness for acid corrosion to create the conditions, so 0–7 days *Alcaligenes* sp. medium than the average corrosion rate of the sterile medium is high, the local corrosion situation is more serious than the sterile medium. At 14 days, corrosion products increased, the corrosion products of the *Alcaligenes* sp. medium and the sterile medium were very different in shape, the surface of X65 steel formed dense clusters of corrosion products. After 21 days, a denser and more uniform film layer was produced on the surface of the carbon steel in the *Alcaligenes* sp. medium compared to the sterile medium, which was considerably more protective.

#### 3.3.2. Analysis of corrosion products

**Figure 4:**
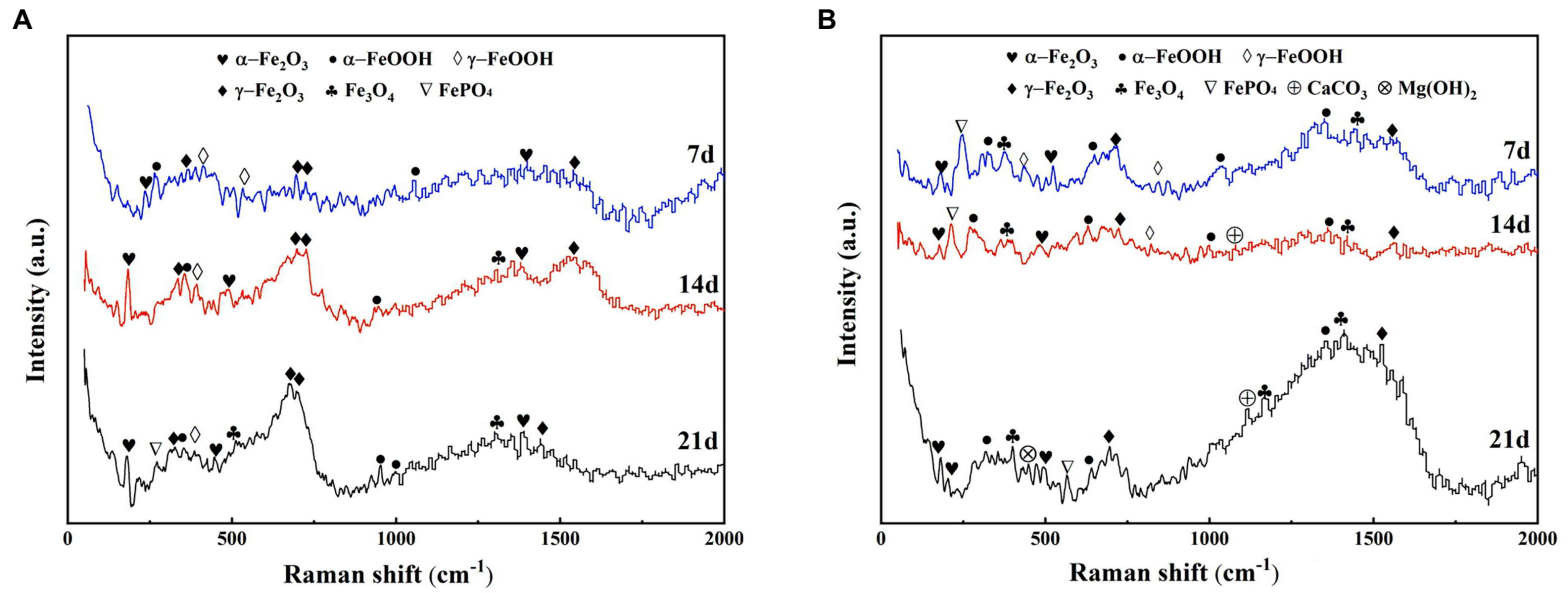
Raman spectra of corrosion products of X65 steel immersed in **(A)** sterile and **(B)** *Alcaligenes* sp. media for different periods.

The compositional changes of the corrosion products of X65 steel at different stages were examined by Raman spectroscopy. Figure 4 shows the Raman spectra of corrosion products of X65 steel after 7, 14, and 21 days of immersion in the sterile and *Alcaligenes* sp. media, respectively. As can be seen from the Figure 4, at different immersion times, the composition and content of corrosion products in the sterile and *Alcaligenes* sp. media are different. This can be seen from Figure 4A, the corrosion product composition of X65 steel was mainly α-Fe_2_O_3_, γ-Fe_2_O_3_, α-FeOOH, γ-FeOOH after 7d immersion in the sterile medium. With the extension of the specimen immersion time (after 14 and 21 days), in addition to the increase in the corrosion product content, the composition of the corrosion products changed, and Fe_3_O_4_ and a small amount of FePO_4_ were detected at 14 and 21 days. After the carbon steel was immersed in the *Alcaligenes* sp. medium for 7 days, the iron oxide content and composition on its surface were different compared with the sterile medium (Figure 4B). In addition to α-Fe_2_O_3_, γ-Fe_2_O_3_, α-FeOOH, and γ-FeOOH, Fe_3_O_4_ and a small amount of phosphate were also detected in the corrosion product composition. After 14d of sample immersion, the content of γ-FeOOH in the *Alcaligenes sp.* medium decreased relative to the sterile medium, while the content of α-FeOOH, Fe_2_O_3_, and Fe_3_O_4_ increased, and phosphate and calcium carbonate components were detected. γ-FeOOH is a corrosion product that is not protective and cannot exist for a long time and is easily converted into stable iron oxides (Navrotsky et al., 2008). By 21 days, γ-FeOOH was not detected in the *Alcaligenes sp.* medium compared to the sterile medium.CaCO_3_ and Mg(OH)_2_ were detected in the medium of *Alcaligenes sp.*α-FeOOH is more stable than γ-FeOOH, the grains are smaller compared to γ-FeOOH, and Fe_2_O_3_ and Fe_3_O_4_ are also stable corrosion products with certain protective properties. With the continuous transformation of γ-FeOOH and the deposition of minerals, the product film becomes more dense and complete, and has a better protective effect on the corrosion of steel.

#### 3.3.3. Biofilm analysis

**Figure 5:**
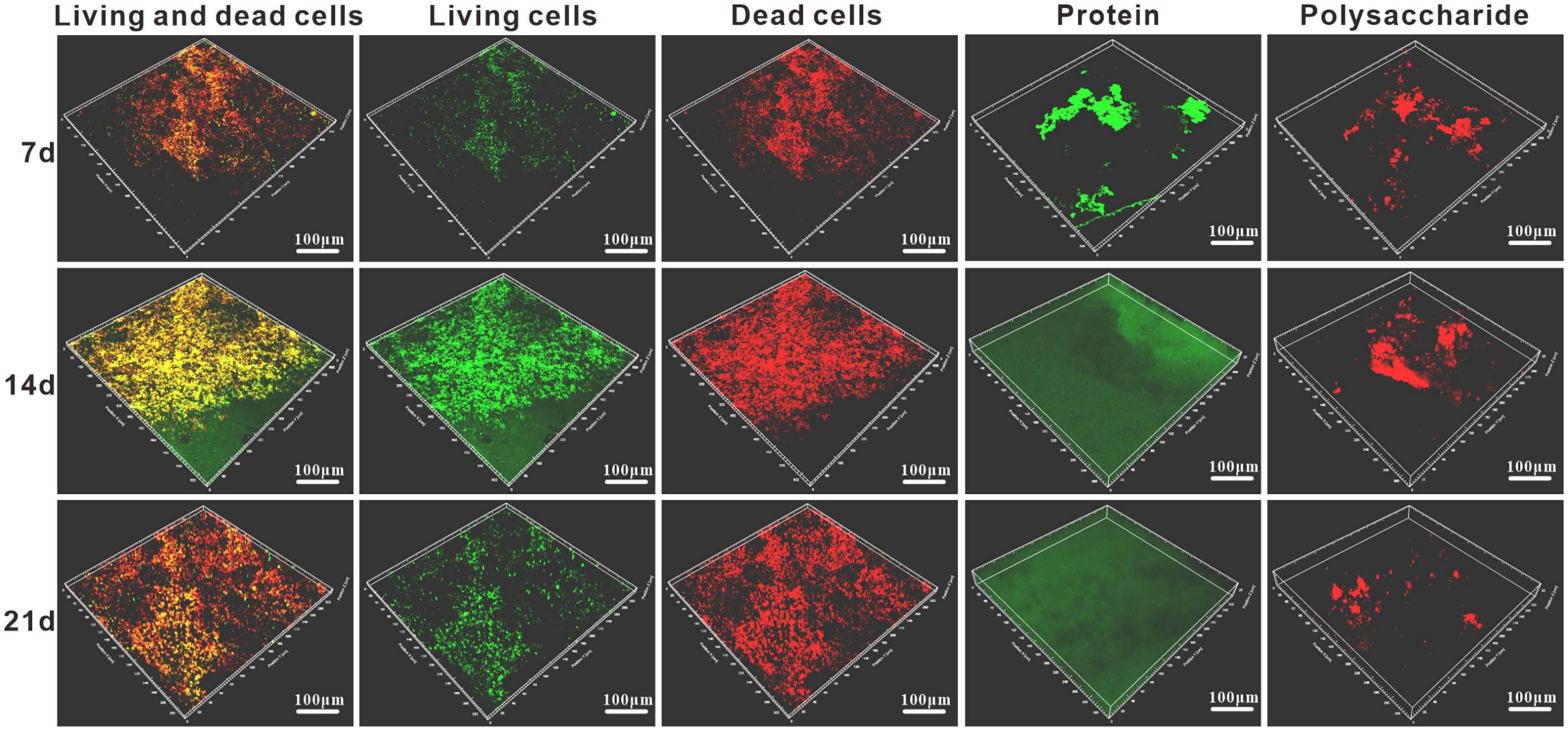
Staining schematic of the live/dead bacteria and protein/polysaccharide in *Alcaligenes* sp. media after different periods of immersion.

The surface biofilm distribution of the samples was analyzed by staining the surface of the specimens with live and dead bacteria and proteoglycans by 3D fluorescence laser confocal microscopy. From Figure 5, it can be found that there were already bacteria attached to the surface of the specimen at 7d, and their distribution was relatively loose with small clustered bacterial aggregates. And the distribution area of dead bacteria on the surface of the specimen was larger than the distribution of live bacteria. Due to the low amount of bacteria attached to the surface of the specimen, the amount of protein and polysaccharides produced by metabolism is low and their distribution on the surface of the specimen is discontinuous. It can be seen that the biofilm structure at this time is smaller and thinner, unevenly distributed and incomplete. The attachment of bacteria on the surface of the specimen increased significantly at 14 days, and the content of live bacteria on the surface of the specimen was higher than that of dead bacteria (Figure 5). With the accumulation of extracellular polymers produced by bacterial growth and metabolism, protein had formed a continuous and complete film layer on the specimen surface, while the content of polysaccharide on the specimen surface did not increase significantly, and its distribution was uneven and discontinuous. Due to the accumulation of bacteria and metabolites, the biofilm was prompted to thicken and form a multilayer biofilm with certain structure. The adsorbed protein layer forms a barrier between the medium and the metal surface, and its role in biofilm development and maturation and in slowing down corrosion is better than that of polysaccharides. The content of live bacteria decreased significantly at 21 days, while the content of dead bacteria did not change significantly (Figure 5). The protein was still uniformly distributed on the surface of the specimen, indicating that the adsorbed film layer of protein was stable and intact. The polysaccharide content decreased, indicating that the adsorption of polysaccharide on the surface of the specimen was unstable and easy to be shed. The biofilm at this time has a tendency to thin.

### 3.4. Analysis of metabolite composition

**Figure 6:**
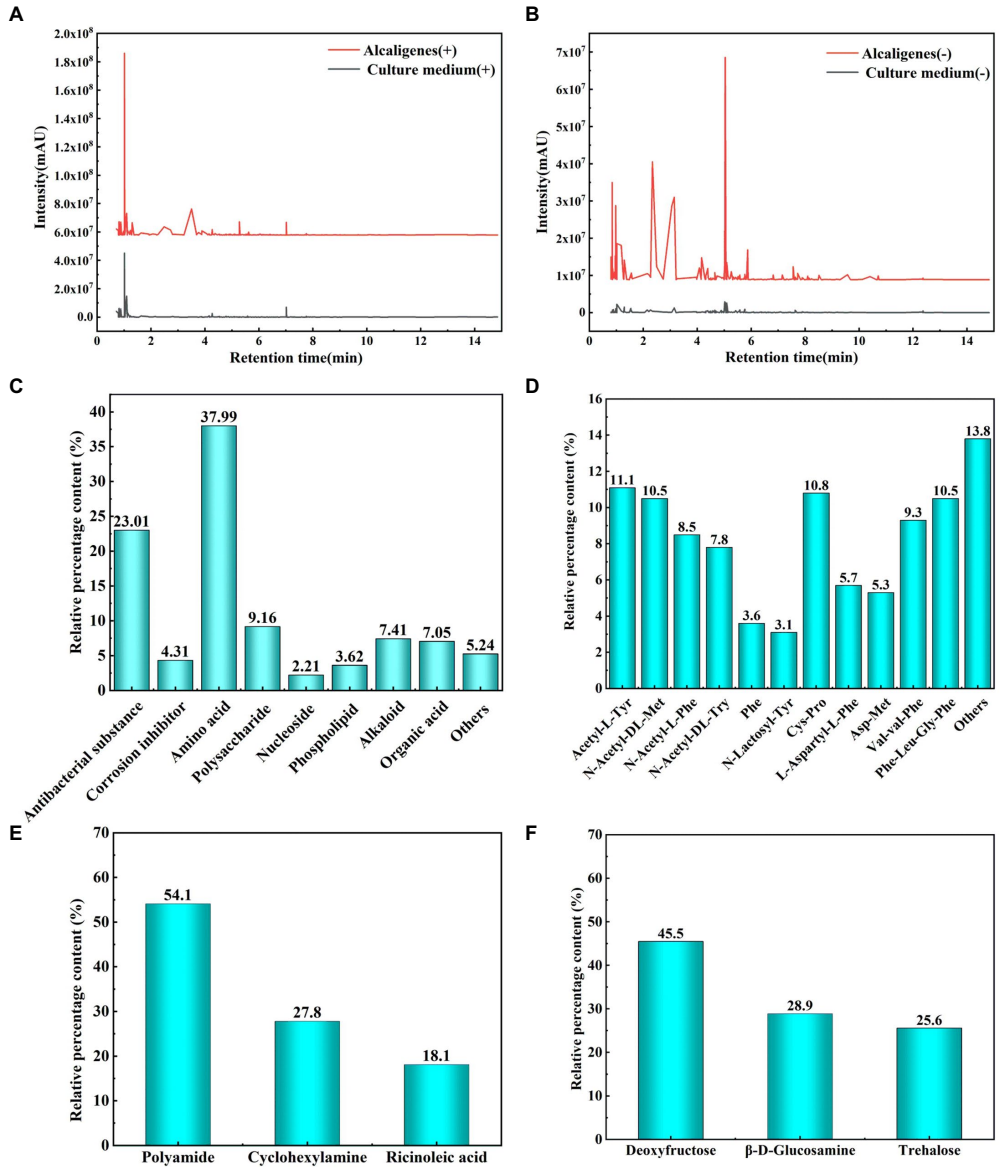
Analysis of metabolites of *Alcaligenes* sp. (**A**: Metabolites correspond to positive ions; **B**: Metabolites correspond to negative ions; **C**: Total metabolites; **D**: Amino acids; **E**: Polysaccharides; **F**: Corrosion inhibitors).

The metabolites were identified using non-targeted metabolomics techniques, grouping different substances according to their different positive and negative ion response, and analyzing the significant increase in metabolism of *Alcaligenes* sp. relative to the medium and the new production of relevant substances by metabolism. Figures 6A,B shows the metabolite profile (positive and negative ion response) of *Alcaligenes* sp. detected by ultra performance liquid chromatography-tandem time-of-flight mass spectrometry. The peak area ratio represents the relative percentage content of each substance in %. Peak area refers to the integral value of peak height. The metabolite composition of *Alcaligenes sp.* is shown in Figure 6C. A total of antibacterial substances (Hydroxyphenyl lactic acid, Phenyl lactic acid, Butane dioic acid, 2,3-Butanediol, Natamycin, Caffeic acid, Glucoside, Gallic acid, Neomycin, Lymecycline, Kanamycin, Ganoderic acid B, Ganoderic acid S, 1,4-Dihydroxyanthraquinone; 23.01%; Zheng et al., 2019, 2020; Wang et al., 2020, 2021a; Abdul-Rahim et al., 2021; Sorgi et al., 2021; Gu et al., 2022; Selvaraj et al., 2022; Wu et al., 2023), corrosion inhibitors (4.31%), amino acids (37.99%), polysaccharides (9.16%), nucleosides (2.21%), phospholipids (3.62%), alkaloids (7.41%), organic acids (7.05%), and other substances (5.24%). The highest relative percentages and the largest variety of substances were amino acids, followed by antibacterial substances. Organic acids are easily soluble in water, while alkaloids are insoluble in water, and the rapid growth of *Alcaligenes* sp. in 1–3 days increases the production of organic acids, so that the pH of the medium drops sharply. The metabolites of *Alcaligenes sp.* had the largest variety of amino acids and the highest relative percentage content, accounting for about 2/5 of the total metabolite amount, which was 4 times that of polysaccharides, resulting in a larger distribution area of proteins than polysaccharides on the surface of the specimen (Figure 5). Proteins and polysaccharides in the extracellular polymers (EPS) secreted by bacteria are the key substances that determine the surface properties of microorganisms, accounting for about 70–80% of the total extracellular polymers(Beech and Sunner, 2004). The composition of amino acids was further analyzed, as shown in Figure 6D. The main types include methionine, cysteine, aromatic amino acids (phenylalanine, tyrosine, tryptophan), and the remaining amino acids include proline, leucine, isoleucine, and arginine, etc. Polysaccharides (Figure 6E) included deoxyfructose, β-D-glucosamine, and alginose, of which the highest content was deoxyfructose, accounting for 45.5% of polysaccharides. Corrosion inhibitors (Figure 6F) include polyamide (Aly et al., 2018), cyclohexylamine(Kumar and Dhanda, 2021), and ricinoleic acid (Zheng et al., 2022), of which the highest content is polyamide, accounting for 54.1% of corrosion inhibitors.

### 3.5. Electrochemical behavior

#### 3.5.1. Changes in open circuit potential

**Figure 7:**
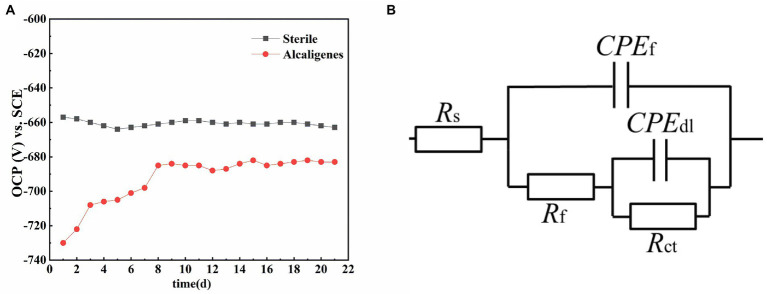
**(A)** Variation of open circuit potential with time for X65 steel in different media. **(B)** Equivalent circuit for simulating electrochemical impedance spectra.

Figure 7A shows the trend of OCP of X65 steel in sterile medium and *Alcaligenes* sp. medium, respectively, over time. As shown in the Figure 7A, the OCP of X65 steel in sterile media remained stable at about −660 mV/SCE. In contrast, the OCP of X65 steel in *Alcaligenes* sp. media fluctuated more significantly, undergoing a process of positive shift and gradual stabilization. This may be due to the accumulation of biofilm and corrosion products, the specimen played a certain protective effect, reducing the tendency of corrosion, which led to the positive shift of OCP in *Alcaligenes* sp. media.

#### 3.5.2. Electrochemical impedance analysis

**Figure 8:**
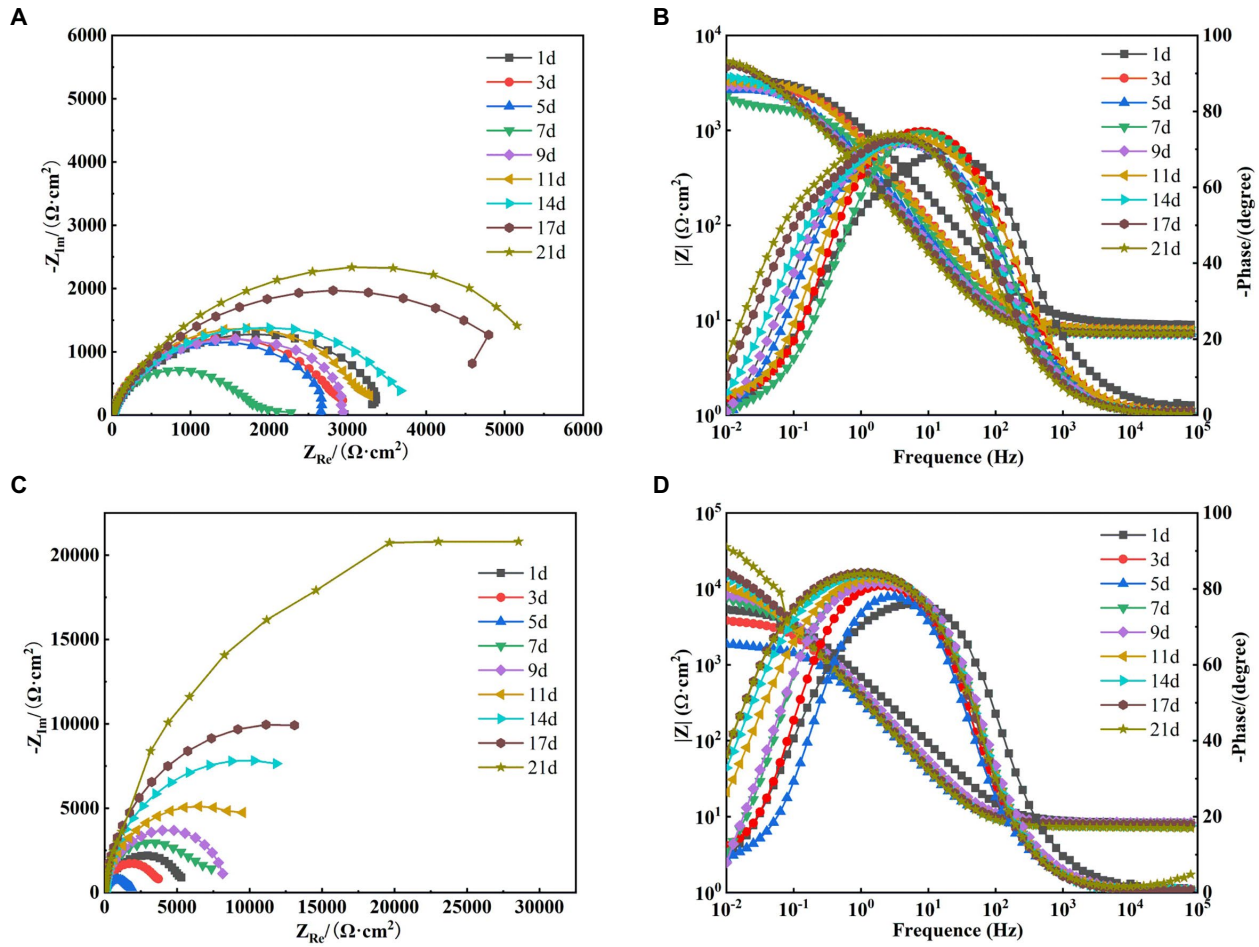
Electrochemical impedance spectra of X65 steel in sterile (**A**: Nyquist spectra; **B**: Bode diagram) and *Alcaligenes* sp. (**C**: Nyquist spectra; **D**: Bode diagram) media.

After the OCP reached steady state, EIS tests were performed on X65 steel working electrodes in different media. Figure 8A shows the Nyquist spectra of X65 steel immersed in sterile media from 1 to 21 days. It can be seen that the radius of capacitive arc resistance decreases gradually at 1–7 days and increases gradually at 7–21 days. This indicates that the corrosion rate of X65 steel in sterile media undergoes a process of increasing and then decreasing. This is probably due to the fact that, with the accumulation of corrosion products, a thicker protective barrier is formed on the surface of X65 steel (Figure 3), which hinders the direct contact between the substrate and the corrosive medium and inhibits the corrosion process. From the Bode diagram can be found (Figure 8B), the total impedance film value shows a trend of first decreasing and then increasing, the phase angle moves to the high frequency region in the early stage and to the low frequency region in the later stage, which also indicates that with the extension of the immersion time, the electrode surface gradually formed a more dense and complete corrosion product film, which can play a certain protective effect on the X65 steel.

Figure 8C shows the Nyquist images of X65 steel immersed in *Alcaligenes* sp. media. It can be seen that the radius of the capacitive arc decreases gradually from 1 to 5 days, and the radius of the capacitive arc increases significantly from 5 to 21 days. This indicates that the corrosion rate of the specimen undergoes a process of first increasing and then decreasing. This is due to the metabolism of organic substances in the medium by *Alcaligenes* sp. to produce organic acids, resulting in a decrease in solution pH, which in turn accelerates corrosion. At the same time due to the initial formation of the film layer of non-uniformity may lead to the metal surface to generate a large number of anode points, and constantly transfer electrons to the nearby cathode area with the presence of oxygen, resulting in the possibility of oxygen corrosion of the material increased. With the adhesion of proteins and polysaccharides on the metal surface and the deposition of minerals, the dense film layer hinders the diffusion of corrosive particles to the steel surface, inhibiting the electrochemical corrosion process of the metal and reducing the corrosion rate. It can be found from the Bode diagram (Figure 8D) that the total impedance film value decreases from 1 to 5 days and increases from 5 to 21 days, which also indicates that the biofilm of *Alcaligenes* sp. and corrosion products complexed on the metal surface to form a more dense and uniform film structure, which enhances the corrosion resistance of X65 steel and makes the corrosion tendency decrease gradually.

In order to better analyze the surface film characteristics and corrosion parameters of X65 steel in *Alcaligenes* sp. media, the impedance data were fitted with a five-element equivalent circuit according to the impedance spectrum characteristics, as shown in Figure 7B. R_s_ is the solution resistance, R_ct_ is the charge transfer resistance, CPE_dl_ is the constant phase angle component of the bilayer, including the bilayer capacitance C_dl_ and dispersion index ndl; R_f_ is the resistance of the film layer attached to the metal surface (including corrosion product film and biofilm), CPE_f_ is the constant phase angle component of the film layer, including the film capacitance *C_f_* and dispersion index n_f_.

The fitted parameters of EIS for X65 steel in different media are shown in Table 1. In the sterile medium, the charge transfer resistance R_ct_ showed a decreasing trend in the first 7d, indicating that the corrosion of X65 steel gradually increased. In the later period, R_ct_ began to increase again, and with the accumulation of corrosion products, the film resistance R_f_ also showed a trend of increasing. Combined with the analysis of the growth curve of *Alcaligenes* sp., it can be seen that the rapid growth and reproduction of *Alcaligenes* sp. in the early stage accelerated the corrosion of X65 steel by metabolizing organic substances into organic acids, resulting in a decrease in the pH of the medium. With the attachment of biofilm and corrosion products on the metal surface, the charge transfer process on the metal surface was hindered, resulting in an increasing charge transfer resistance of 5–21 days, while the film resistance R_f_ and film capacitance *C_f_* were also increasing, and the dispersion index was also larger, indicating that with the gradual formation of a more dense and complete product film on the surface of the specimen, which prevented the active dissolution of Fe on the metal surface and played a certain protective role for the specimen. Throughout the experiment, the R_f_ value of the *Alcaligenes* sp. medium is basically greater than the value of the corresponding time of the sterile medium, indicating that the physical barrier effect of the mixed film layer of *Alcaligenes* sp. and its metabolic products complexed with corrosion products accumulated on the surface of the specimen is greater than that of the corrosion products of Fe alone.

**Table 1:** AC impedance spectrum fitting parameters for X65 steel in different media.

	T(d)	R_s_ (Ω cm^2^)	R_ct_ (Ω cm^2^)	C_dl_ (F cm^2^)	n_dl_	R_f_ (Ω cm^2^)	*C_f_* (F cm^2^)	n_f_
Sterile	1	9.14	3,384	1.91 × 10^−4^	0.83	181.29	1.45 × 10^−4^	0.88
	3	7.92	2,941	2.09 × 10^−4^	0.88	124.99	2.12 × 10^−4^	0.89
	5	7.19	2,707	3.85 × 10^−4^	0.89	82.09	4.03 × 10^−4^	0.88
	7	8.00	1868	2.70 × 10^−4^	0.86	81.84	2.55 × 10^−4^	0.91
	9	7.18	3,016	4.65 × 10^−4^	0.87	158.85	4.64 × 10^−4^	0.90
	11	7.85	3,269	2.37 × 10^−4^	0.89	180.51	5.53 × 10^−4^	0.89
	14	7.09	3,764	5.23 × 10^−4^	0.83	261.21	5.83 × 10^−4^	0.90
	17	7.26	5,413	5.51 × 10^−4^	0.81	280.58	8.93 × 10^−4^	0.87
	21	7.31	6,288	6.16 × 10^−4^	0.81	353.84	9.62 × 10^−4^	0.87
*Alcaligenes*	1	8.40	5,692	2.93 × 10^−4^	0.83	252.70	5.57 × 10^−4^	0.92
	3	8.28	3,942	4.53 × 10^−4^	0.91	175.29	5.05 × 10^−4^	0.93
	5	8.30	2,689	2.64 × 10^−4^	0.69	166.77	4.70 × 10^−4^	0.94
	7	7.75	7,002	4.12 × 10^−4^	0.93	175.79	4.38 × 10^−4^	0.95
	9	8.38	8,325	3.66 × 10^−4^	0.94	309.85	6.76 × 10^−4^	0.94
	11	7.72	12,975	4.42 × 10^−4^	0.86	422.11	8.58 × 10^−4^	0.95
	14	7.70	19,159	4.41 × 10^−4^	0.87	478.20	1.50 × 10^−3^	0.95
	17	8.10	24,261	4.04 × 10^−4^	0.88	612.58	3.48 × 10^−3^	0.96
	21	7.25	41,184	2.90 × 10^−4^	0.99	815.99	3.90 × 10^−3^	0.96

#### 3.5.3. Polarization curve analysis

**Figure 9:**
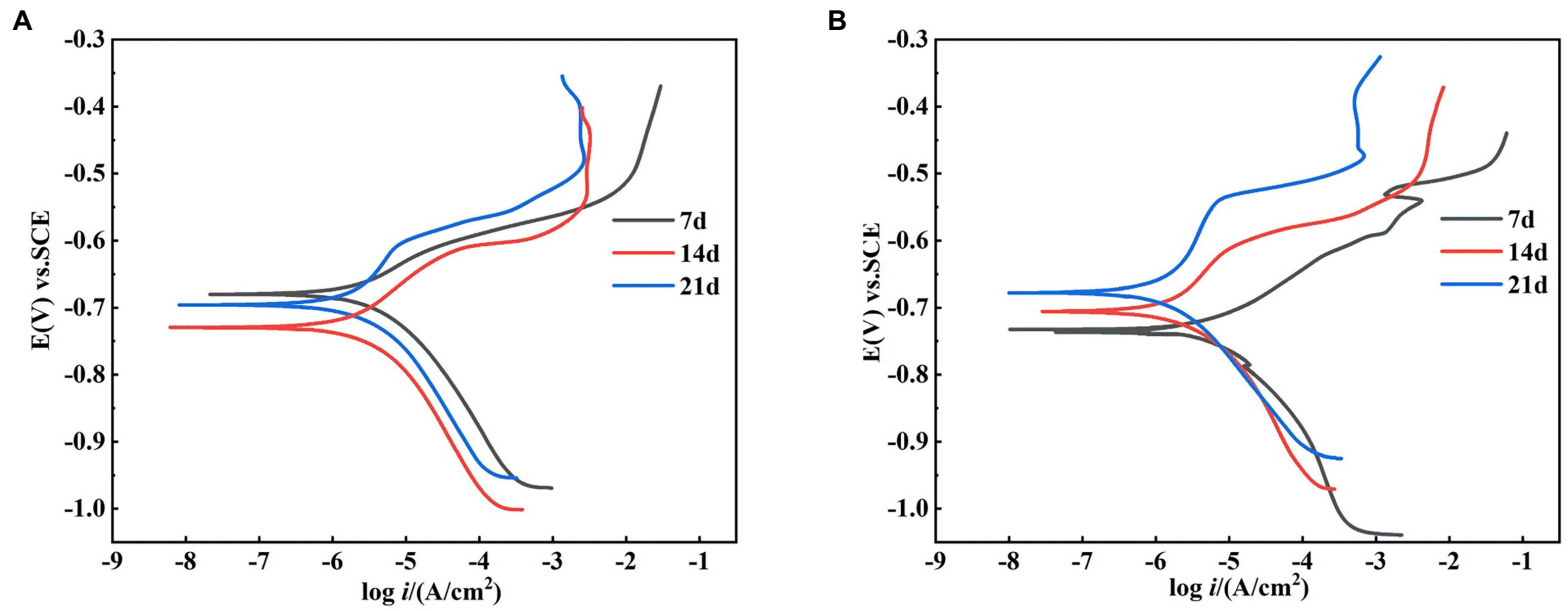
Kinetic potential polarization curves of X65 steel after 7, 14, and 21 d of polarization in **(A)** sterile and **(B)** *Alcaligenes* sp. media.

The dynamic potential polarization curve is an important method to reveal the corrosion mechanism of metals, which can not only determine the type of corrosion reaction and the ease of reaction according to the trend of cathodic and anodic processes, but also determine the corrosion rate of metals. Therefore, this experiment tested the dynamic potential polarization curve of X65 steel in sterile and *Alcaligenes* sp. medium after 7, 14, and 21 days immersion respectively, as shown in Figure 9, and its corresponding fitting results are shown in Table 2. Where β_a_ and β_c_ are the Tafel slopes of the anodic and cathodic processes, respectively, and E_corr_ and i_corr_ are the corrosion potential and corrosion current density, respectively. In the sterile medium, it is known from the fitted parameters that the corrosion current densities of 7, 14 and 21 days are 2.56 × 10^−6^, 2.46 × 10^−6^, and 1.78 × 10^−6^A/cm^2^, respectively, and the corrosion rate of X65 steel decreases. The anodic Tafel slope at 7d is smaller than the cathodic Tafel slope, indicating that the contact between the specimen immersed in the sterile medium and the corrosive medium in the early stage makes the metal surface undergo faster anodic dissolution (Fe → Fe^2+^ + 2e^−^) and the anodic corrosion rate is larger. The shapes of the polarization curves at 14 and 21 days were similar, indicating that the cathodic-anodic reaction histories did not change significantly, and the kinetic laws of X65 steel at 14–21 days were basically the same. In the *Alcaligenes* sp. medium, the shape of the polarization curve changed significantly with increasing immersion time. This implies that the corrosion process of X65 steel is closely related to the microbial activity, and the bacterial growth metabolism is involved in and influences the electron transfer process on the metal surface, thus affecting the reaction kinetics of both cathodic and anodic processes. The increase in cathodic and anodic Tafel slope is due to the fact that the faster dissolving Fe^2+^ requires a large amount of OH^−^ produced by the cathode to combine to form hydroxide and oxide of Fe, the corrosion products on the surface of the specimen accumulation, so that the dissolution of iron and the depolarization of oxygen are blocked by the mass transfer process. Compared with the sterile medium, the i_corr_ of 7d was greater than that of the sterile medium, and the slope of the anode Tafel was smaller than that of the sterile medium, which was due to the organic acid produced by the metabolism of *Alcaligenes* sp. that played a facilitating role in the corrosion of X65 steel. i_corr_ of 14d and 21d was smaller than that of the sterile medium, and the slope of the anode Tafel was larger than that of the sterile medium. This is due to the fact that the produced proteoglycans and corrosion inhibiting substances contain a large number of polar groups, which complex with iron ions under pH-stable conditions and form insoluble complexes that enhance the stability of the film layer. With the transformation of unstable corrosion products to stable corrosion products and the deposition of *Alcaligenes* sp.-induced minerals, the structure of the product film layer is uniformly and continuously distributed on the surface of the specimen (Figure 3), making the corrosion rate decrease.

**Table 2:** Fitting parameters of the kinetic potential polarization curves of X65 steel in different media.

	T(d)	*E*_corr_ (V) vs. SCE	*I*_corr_ (A/cm^2^)	β_a_ (mV/dec)	β_c_ (mV/dec)
Sterile	7	−0.680	2.56 × 10^−6^	87.84	114.50
	14	−0.729	2.46 × 10^−6^	99.71	119.70
	21	−0.696	1.78 × 10^−6^	155.24	138.38
*Alcaligenes*	7	−0.733	2.90 × 10^−6^	72.94	109.50
	14	−0.706	2.13 × 10^−6^	126.60	132.15
	21	−0.678	5.59 × 10^−7^	246.54	162.18

## 4. Discussion

**Figure 10:**
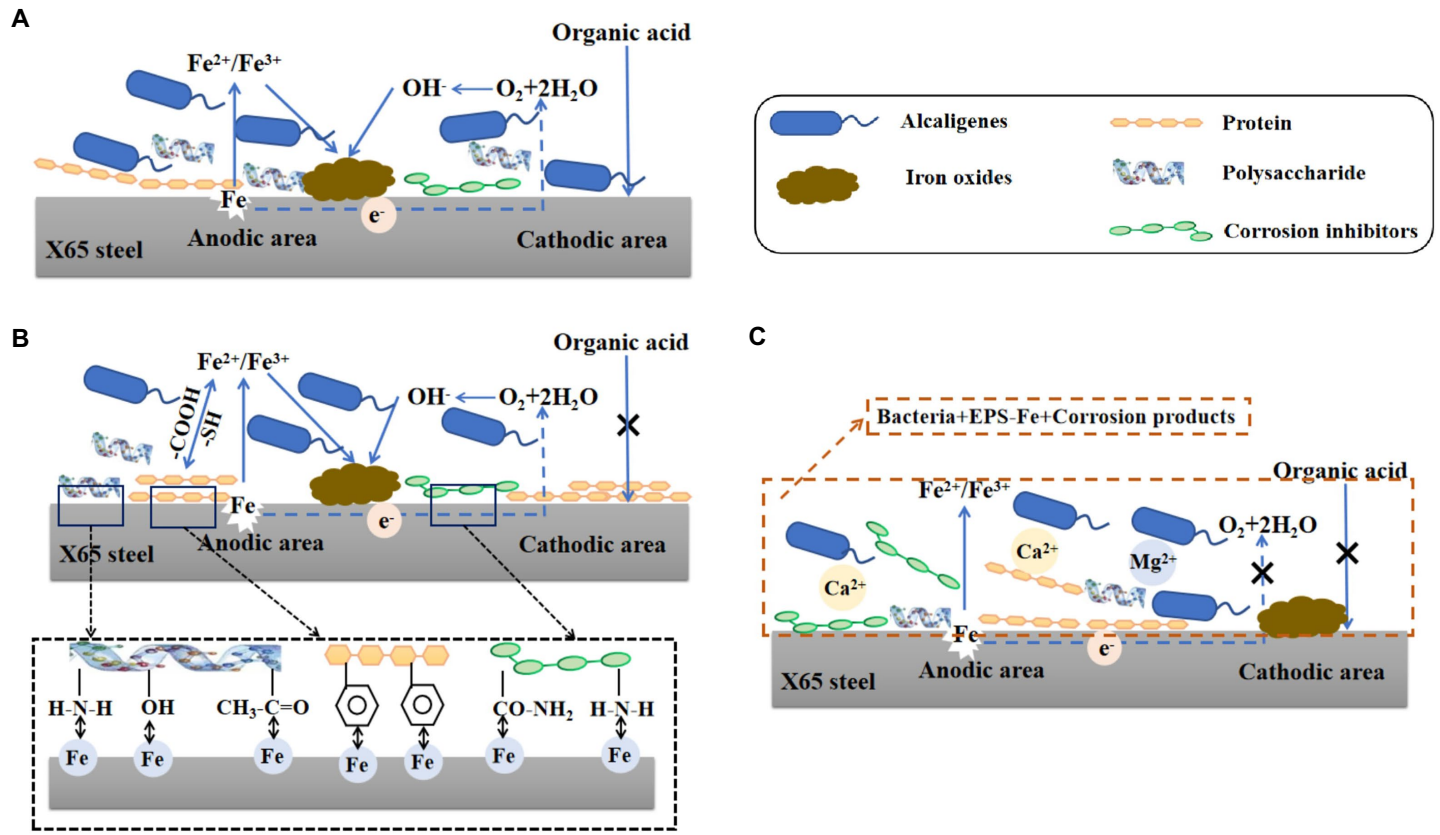
Schematic diagram of the modeled corrosion mechanism of X65 steel by *Alcaligenes* sp. in different periods.

Through the above study, the mechanism of corrosion of X65 steel by *Alcaligenes sp.* was explored and the mechanism is shown schematically in Figure 10. As shown in Figure 10A, at the early stage of corrosion reaction (1–7 days), *Alcaligenes* sp. began to adhere to the surface of carbon steel and secreted EPS to provide space for their growth. According to the bacterial count, the amount of planktonic bacteria is 1–3 orders of magnitude higher than the amount of sessile bacteria, and compared with planktonic bacteria, the growth period of sessile bacteria is longer and the highest bacterial counts appeared later. From the biofilm analysis, the distribution area of protein and polysaccharide on the surface of the 7 days specimen was comparable and both showed a zonal pattern, while the protein content produced by *Alcaligenes* sp. was much higher than that of polysaccharide, indicating that polysaccharide was preferentially adsorbed on the metal surface. From Raman analysis, it is clear that the unstable corrosion products (γ-FeOOH) formed on the metal surface are more and loosely distributed. So the biofilm and corrosion products formed during this period are not uniform and incomplete, leading to the formation of an oxygen concentration differential cell, while creating the conditions for organic acid corrosion. Compared with the sterile medium, the charge transfer resistance of this time period is reduced, the average corrosion rate is high, and the immobility of the active anode site location leads to the formation of a large anodic area and uniform corrosion occurs.

As shown in Figure 10B, in the middle of the corrosion reaction (7–14 days), the amount of planktonic bacteria decreased, and the number of sessile bacteria was relatively stable and stable for a longer period, 10–100 times higher than the amount of planktonic bacteria, indicating that the biofilm provided a stable growth environment for bacterial growth. Compared with the sterile medium, the impedance film value of the *Alcaligenes* sp. medium increased, the cathodic slope increased and the corrosion current density decreased in this period. This is due to the gradual formation of a continuous and complete film layer of proteins on the metal surface. From the analysis of the metabolites, it is clear that amino acids account for the largest proportion of substances, of which the main types are methionine, cysteine and aromatic amino acids. The corrosion inhibition efficiency of amino acids on carbon steel in acidic medium is related to the lowest orbital energy level of amino acids, the lower the lowest orbital energy level, the higher the corrosion inhibition efficiency. This is because when amino acids produce corrosion inhibition at the carbon-steel-interface, the highest occupied orbital at the carbon-steel-interface interacts with the lowest vacant orbital of amino acids, and electron transfer occurs, resulting in chemisorption, so that amino acid molecules are absorbed at the carbon-steel-interface, separating H^+^ from the carbon-steel-interface, thus achieving corrosion inhibition (Wang et al., 2021b). The – COOH is a functional group for complexing metal ions in EPS, which contains C = O and C – O can complex with iron ions or other metal ions favoring the formation of a dense protective layer (Ghafari et al., 2013). Methionine and cysteine belong to sulfur-containing Amino acids, and – SH is the most polar group among Amino acids that can easily bind metal ions, the hydrogen ion on – SH can be free in water, while its negative ion combines with metal ions to form a very strong complex protective film, and this very tight protective layer prevents further metal erosion (Abdel-Fatah et al., 2014). The electron clouds on the aromatic rings of aromatic Amino acids (phe, tyr, and try) and the lone pairs of electrons on the heteroatoms interact with the empty orbitals of iron to form insoluble complexes that cover the metal surface, while the hydrophobic benzene rings form a directional upward arrangement that separates the corrosive medium from the surface of X65 steel, thus inhibiting the corrosion (Ashassi-Sorkhabi et al., 2005; Zhang et al., 2008). Polysaccharides can effectively adsorb on the metal surface to form a passivation protective layer blocking the attack of corrosive ions on the metal because they contain polar functional groups such as –NH_2_, –OH and acetyl groups, and secondly, C, N and O atoms in polysaccharides can provide electrons for the empty orbitals of iron to form stable complexes and inhibit corrosion reactions at the cathode and anode (An and Friedman, 1998). The metabolites such as corrosion inhibitors produced have a large number of functional adsorption centers such as – NH_2_, – CO – NH_2_, and O, N heteroat (Gece, 2011). Also, the presence of long hydrophobic alkyl chains can effectively improve the hydrophobicity of the adsorbed membrane (Mobin et al., 2016). In contrast, there was no significant increase in the content of polysaccharides on the surface of the specimen, and their distribution was uneven and discontinuous due to the small content of polysaccharides produced by *Alcaligenes* sp., which was 1/4 of the protein content, and the polysaccharides produced contained – NH_2_ and – OH, which showed relatively weak complexation ability compared to – COOH and – SH. The biofilm of this period is dominated by proteins and bacteria, in which polysaccharides are embedded, and the adsorbed protein layer is closer to the substrate of the specimen, forming a barrier between the medium and the metal surface, and its role in biofilm development and maturation and in slowing down corrosion is better than that of polysaccharides.

As shown in Figure 10C, in the late stage of corrosion reaction (14–21 days), the amount of planktonic bacteria and the amount of solidified bacteria both decreased, but the amount of planktonic bacteria decreased more than the amount of solidified bacteria. A complete dense protective film with a certain thickness was formed on the metal surface. On the one hand, this is because the biofilm is thinned, but the adsorbed protein layer still forms a film with uniform thickness. The insoluble complexes formed by the polar groups in the proteins and the iron ions under the conditions of pH stability strengthen the stability of the film. On the other hand, the presence of *Alcaligenes* sp. promotes the generation of stable corrosion products Fe_2_O_3_ and Fe_3_O_4_, and EPS is an ideal nucleation site for mineral deposition, which can be used as a binding site for Ca^2+^and Mg^2+^, leading to the deposition of calcite structured CaCO_3_ and Mg (OH)_2_ minerals (Spadafora et al., 2010; Perri et al., 2022). Therefore, the impedance film value of the *Alcaligenes* sp. medium in this period further increases and is higher than that of the sterile medium, and the cathodic slope further increases and the corrosion reaction is inhibited.

## 5. Conclusion

In the early stage (0–7 days), the low content and loose distribution of biofilm and corrosion products on the carbon steel surface led to the formation of oxygen concentration difference cells, while organic acids produced by *Alcaligenes* sp. during growth and metabolism lowered the pH of the environment, and the organic acids accelerated the dissolution of Fe^2+^ when they came in contact with the metal surface. In the middle and late stages (7–21 days), proteoglycans and corrosion inhibiting substances produced by *Alcaligenes* sp. were enriched on the metal surface, and with the formation of stable corrosion products and the deposition of induced minerals, the stability of the film layer was enhanced, and the corrosion rate of X65 steel was greatly reduced and local corrosion was inhibited. This study broadens the application scope of *Alcaligenes sp.* and provides theoretical guidance for its application in the field of metal corrosion inhibition.

## Data availability statement

The original contributions presented in the study are included in the article/supplementary material, further inquiries can be directed to the corresponding author.

## Author contributions

PS: conceptualization, investigation, data curation, writing-original draft. MD: funding acquisition, resources, supervision, writing—review and editing. JW: methodology, data curation, writing—review and editing. All authors contributed to the article and approved the submitted version.

## Funding

This work was financially supported by the National Nature Science Foundation of China (nos. 52071302 and 51871204).

## Conflict of interest

The authors declare that the research was conducted in the absence of any commercial or financial relationships that could be construed as a potential conflict of interest.

## Publisher’s note

All claims expressed in this article are solely those of the authors and do not necessarily represent those of their affiliated organizations, or those of the publisher, the editors and the reviewers. Any product that may be evaluated in this article, or claim that may be made by its manufacturer, is not guaranteed or endorsed by the publisher.
